# Ca^2+ ^buffering as a mechanism of short-term synaptic plasticity

**DOI:** 10.1186/1471-2202-14-S1-P269

**Published:** 2013-07-08

**Authors:** Victor Matveev

**Affiliations:** 1 Department of Mathematical Sciences, New Jersey Institute of Technology, Newark, NJ 07102, USA

## 

**Figure 1:**
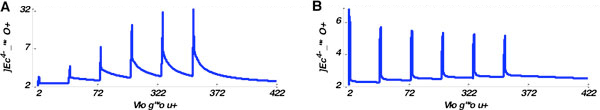
**[Ca^2+^] elevation during a 40 Hz train of 1ms-long Ca^2+ ^pulses at 100nm from the Ca^2+ ^source**. **A: **In the presence of a *non-cooperative *buffer, Ca^2+ ^transients facilitate due to gradual depletion (saturation) of free buffer [6,7]. **B: **In the presence of a *cooperative *buffer, Ca^2+ ^transients depress as a result of increasing exposure of the high-affinity Ca^2+ ^binding site. Both simulations done in 1D geometry (0.5 μm axonal segment), computed using CalC version 7.2 (http://www.calciumcalculator.org)

Spatio-temporal compartmentalization allows Ca^2+ ^signals to simultaneously regulate multiple vital cell processes and relies in part on Ca^2+ ^buffers that absorb at least 98% of Ca^2+ ^ions entering the cytoplasm. Computational modeling has played a central role in the understanding of localized Ca^2+ ^signals in neurons and other cell types. Although many models consider only simple, one-to-one Ca^2+ ^buffering stoichiometry, practically all buffers have multiple Ca^2+ ^binding sites which often display cooperative binding (e.g., calretinin [1-3], calmodulin [4,5] ). Given the simplest case of two binding sites, cooperativity manifests itself in an order of magnitude difference in the binding and/or unbinding rates of the two consecutive Ca^2+ ^binding steps in the following buffer reaction:

Here we extend recent modeling studies of cooperative buffering [1-5], and find that it can lead to spatio-temporal Ca^2+ ^signals that cannot be achieved by any combination of non-cooperative buffers, in particular during a sequence of action potentials or synaptic inputs. Namely, Figure 1B shows that cooperative Ca^2+ ^buffering can potentially serve as a mechanism of short-term synaptic depression, in contrast to the case of non-cooperative buffers (Figure 1A), which are believed to underlie short-term synaptic facilitation in certain types of mammalian synapses [6,7].

We explore this phenomenon in detail, demonstrating the dependence of such buffer-induced short-term synaptic plasticity on all relevant buffering parameters. These results may lead to better understanding of *post*-synaptic Ca^2+ ^dynamics as well, yielding a deeper insight into synaptic transmission and its dynamic regulation, and may also have relevance for Ca^2+^-dependent processes in other cells such as endocrine cells, myocytes and immune system cells.
